# The effects of the bacterial interaction with visible-light responsive titania photocatalyst on the bactericidal performance

**DOI:** 10.1186/1423-0127-16-7

**Published:** 2009-01-15

**Authors:** Chia-Liang Cheng, Der-Shan Sun, Wen-Chen Chu, Yao-Hsuan Tseng, Han-Chen Ho, Jia-Bin Wang, Pei-Hua Chung, Jiann-Hwa Chen, Pei-Jane Tsai, Nien-Tsung Lin, Mei-Shiuan Yu, Hsin-Hou Chang

**Affiliations:** 1 Department of Physics, National Dong-Hwa University, Hualien, Taiwan; 2 Nanotechnology Research Center, National Dong-Hwa University, Hualien, Taiwan; 3 Institute of Molecular Biology and Human Genetics, Tzu-Chi University, Hualien, Taiwan; 4 Institute of Medical Science, Tzu-Chi University, Hualien, Taiwan; 5 Department of Life Science, Tzu-Chi University, Hualien, Taiwan; 6 Department of Chemical Engineering, National Taiwan University of Science and Technology, Taipei, Taiwan; 7 Department of Anatomy, Tzu-Chi University, Hualien, Taiwan; 8 Institute of Molecular Biology, National Chung Hsing University, Taichung, Taiwan; 9 Institute of Medical Biotechnology, Tzu-Chi University, Hualien, Taiwan; 10 Institute of Microbiology, Immunology and Molecular Medicine, Tzu-Chi University, Hualien, Taiwan

## Abstract

Bactericidal activity of traditional titanium dioxide (TiO_2_) photocatalyst is effective only upon irradiation by ultraviolet light, which restricts the potential applications of TiO_2 _for use in our living environments. Recently carbon-containing TiO_2 _was found to be photoactive at visible-light illumination that affords the potential to overcome this problem; although, the bactericidal activity of these photocatalysts is relatively lower than conventional disinfectants. Evidenced from scanning electron microscopy and confocal Raman spectral mapping analysis, we found the interaction with bacteria was significantly enhanced in these anatase/rutile mixed-phase carbon-containing TiO_2_. Bacteria-killing experiments indicate that a significantly higher proportion of all tested pathogens including *Staphylococcus aureus*, *Shigella flexneri *and *Acinetobacter baumannii*, were eliminated by the new nanoparticle with higher bacterial interaction property. These findings suggest the created materials with high bacterial interaction ability might be a useful strategy to improve the antimicrobial activity of visible-light-activated TiO_2_.

## Background

The widespread use of antibiotics and the emergence of more resistant and virulent strains of microorganisms [1-3] have caused an urgent need to develop alternative sterilization technologies. Using the superb photocatalytic effect of titanium dioxide (TiO_2_) is a conceptually feasible technology for this material is easy and inexpensive to produce in industrial scale. Photocatalytic TiO_2 _substrates have been shown to eliminate organic compounds and to function as disinfectants [4]. Upon ultraviolet (UV) light excitation, the photon energy excites valence band electron and generates pairs of electrons and holes (electron-vacancy in valence band) that diffuse and are trapped on or near the TiO_2 _surface. These excited electrons and holes have strong reducing and oxidizing activity and react with atmospheric water and oxygen to yield reactive species such as hydroxyl radicals (^.^OH) and superoxide anions (O_2_^-^) [5]. These radicals, ^.^OH and O_2_^- ^are extremely reactive upon contact with organic compounds. Complete oxidation of organic compounds and bacterial cells to carbon dioxide could be achieved [6,7]. Reactive oxygen species (ROS), such as ^.^OH, O_2_^-^, and hydrogen peroxide (H_2_O_2_) generated on the light irradiated TiO_2 _surfaces, were shown to operate in concert to attack polyunsaturated phospholipids in bacteria [4]. Traditional TiO_2 _photocatalyst, however, is effective only upon irradiation of UV-light at levels that would also induce serious damage to human cells. This greatly restricts the potential applications of TiO_2 _substrates for use in our living environments. Recently, nitrogen or metal ion-doped anatase based TiO_2 _photocatalysts have been identified to be active upon visible-light illumination [8,9], offering the possibility to overcome this problem.

It is believed that nanometer-sized anatase phase particles have large surface area are efficient for the decomposition of pollutants in air and in water [10]. Furthermore, it is also found that the presence of anatase and rutile phases is important in some of the photocatalytic reactions where oxygen is used as electron acceptor [10]. Transmission electron microscopy studies also revealed that commercial TiO2 powder Degussa (P-25) consisting both anatase and rutile phases [11]. However, in these studies, the photocatalytic activities were induced under UV irradiation (wavelength < 380 nm). Previously, we have produced carbon-containing TiO_2 _in two different calcination temperatures (150°C and 200°C) resulted in two different nano-crystals (labeled as C150 and C200, respectively) with photocatalytic activity in the visible-light range [12]. These materials seem to be more convenient to apply in our living environment than the commercial UV responsive photocatalysts. The antibacterial activity of visible-light responsive photocatalysts has been reported by several groups [13-15]. Since photocatalyst-based anti-microbial technologies are still under development, the antibacterial activity of these materials does not match to that of conventional chemical disinfectants [13,16]. To improve the antibacterial activity, previous studies were mainly focused on the photocatalysis properties [17,18], while the photocatalyst-bacterial interactions were rarely discussed.

In this present study, scaning electron microscopy and confocal Raman spectrscopy were used to study different photocatalysts interact with pathogens. The photocatalyst-bacterial interaction properties were then compared to the bactericidal activity of respective photocatalysts. To further investigate whether the antibacterial effect can be generally applied to human pathogens, we tested several human pathogens including *Staphylococcus aureus*, *Shigella flexneri *and *Acinetobacter baumannii*. Among these bacteria, *S. flexneri *is a food-borne pathogen, which is usually found in contaminated water, plants, and sewage [19-22], and frequently leads to outbreaks in regions with poor sanitary conditions [21,23]. *S. aureus* is a exotoxin producing pathogen which can cause diseases such as food-borne diseases, soft tissue infections, and toxic shock syndrome in humans[19]. The emergence and rapid spread of multidrug-resistant *A. baumannii *isolates causing nosocomial infections are of great concern worldwide [24]. The antimicrobial performance of the visible-light responsive titania catalysts against these bacteria will be compared.

## Materials and methods

### Preparation of TiO_2_, C150 and C200 nanoparticles

Carbon-containing mixed phase nano-structured TiO_2 _powders were prepared using a modified sol-gel method. The produced powders were subjected to calcination at 150°C and 200°C, and named as C150 and C200, respectively. Details in preparation of C150 and C200, structural properties, the sizes of primary particles, light absorption, etc. have been reported elsewhere [12]. In our previous study [12], we found the C200 has a unique anatase/rutile mixed crystalline phases that exhibits strong visible-light absorption and photocatalytic effects. The photocatalytic studies have been reported previously [12,25,26]. In these TiO_2_, carbons exist in an amorphous form as seen in the Raman spectra, and the carbon contents were estimated using x-ray photoelectron spectroscopy to be approximately ~30 atomic % on the surface (data not shown). One commercially available TiO_2 _nanopowder (UV100, Sachtleben, Germany), that can exert the photocatalytic property only when illuminated by UV light, was used for comparison. Since C150 and C200 samples often aggregate into larger cluster due to surface charges, Van der Waals interactions, we dispersed the aggregates using sonication (Transsonic digital TP680DH, Ultrasonic cleaning Co. Singapore, Singapore) before the bacteria-killing or bacteria-photocatalyst interaction experiments.

### Confocal Raman spectral mapping

Confocal Raman mapping was carried out with a confocal Raman spectrometer using 488 nm excitation wavelength (α-SNOM, Witec, Germany). The confocal Raman mapping has a spatial resolution of ~250 nm; typical scan were performed in an area of 10 × 10 μm^2 ^area and in air. The mapping consisted of 0.2 μm in each step in both the x and y directions, with specific Raman signals of the interested sample components are plotted to form a 2-D map to reveal the structural distribution of the interested structures. The bacteria-nanoparticle images were taken after 20 μl of nanoparticles (10 mg/ml) and bacteria (1 × 10^6 ^CFU/ml) suspensions in H_2_O were spread on cover glasses and dry. Laser power were kept low (less than 1 mW) to avoid damaging the test samples, both the TiO_2 _and the bacteria.

### Scanning electron microscopic imaging

Scanning electron microscopic (SEM) analysis was performed as previously described [27-30]. The images were obtained using a JEM-3010 scanning electron microscope (JEOL, Japan) equipped with energy dispersive x-ray spectrometer (EDS) for the chemical elemental analysis to observe the surface morphology of the tested TiO_2 _nanoparticles. To observe the interaction of microbes and TiO_2 _samples, bacteria and TiO_2 _powders were mixed and subjected to photocatalytic reaction as described in next sections. After the reaction, the samples were transferred to cover-glasses and fixed by 2.5% glutaraldehyde in 0.1 M phosphate buffer, then 1% osmium tetroxide in 0.1 M phosphate buffer, pH 7.3, and then subjected to a series of alcohol dehydration, critical point drying procedures, and gold coating [27] and observed under a scanning electron microscope at 15 kV (Hitachi S-4700, Hitachi, Japan). At least three different areas were randomly selected for photography at each magnification; representative data are shown.

### Bacterial strains and culture

Basic bacterial cultural methods were performed as previously described [13,31]. Clinical isolated *S. flexneri *was collected from a shigellosis outbreak in central Taiwan in 1996 [23]. *A. baumannii*, pan-drug resistant *A. baumannii *and *S. aureus *were clinical isolates from Buddhist Tzu-Chi General Hospital in Hualien, Taiwan. All isolates were initially differentiated into Gram positive and Gram-negative strains by a standard staining procedure. The bacteria were cultured in tryptic soy broth supplemented with 0.5% yeast extract (TSBY) and LB at 37°C for 16 hr, and then identified by biochemical methods according to routine clinical laboratory procedures [32]. *S. flexneri*, *A. baumannii *and pan-drug resistant *A. baumannii *were maintained and grown in LB medium or LB agar at 37°C. Bacterium *S. aureus *was grown in TSBY broth or TSBY broth agar (MDBio, Inc. Taipei, Taiwan) at 37°C. All bacteria isolates were stored in 50% glycerol (V/V) in culture medium at -80°C before use. To reactivate bacteria from frozen stocks, 25 μl bacterial stock solution was transferred to a test tube containing 5 ml of freshly prepared culture medium and then incubated at 37°C under agitation overnight (16–18 hr).

### Bactericidal effects of the TiO_2 _nanoparticles

In this study, bacterial concentrations were either determined by the standard plating method or inferred from optical density readings at 600 nm (OD_600_). For each bacterium, a factor for converting the OD_600_values of the bacterial culture to concentration (CFU/ml) was calculated as the followings. A fresh bacterial culture was diluted by factors of 10^-1 ^to 10^-7^, and OD_600 _of these dilutions was measured. Bacterial concentrations of these dilutions were determined using standard plating method. The OD_600 _values were plotted against the bacterial concentrations' log values, and the conversion factors for particular bacteria were calculated. The conversion factor for *S. aureus*, for example, was calculated to be 1 × 10^8 ^CFU/ml per OD_600 _by this method.

In order to determine the bactericidal effects of the TiO_2 _nanoparticles, 200 μl of bacterial overnight culture was transferred into 5 ml of culture medium and incubated at 37°C until an OD_600 _of 0.3 to 0.6 (log phase) was reached. The bacterial concentrations were calculated using the conversion factor for the bacteria, and the cultures were diluted to 5 × 10^5 ^CFU/ml with culture medium. Fifty micro liters of the bacterial culture (2.5 × 10^4 ^CFU) were mixed with the TiO_2 _nanoparticles (200 μg/ml in 150 μl normal saline) using a plastic yellow tip and placed onto a 24-well cell culture dish. The cell culture dish was then placed under an incandescent lamp (Classictone incandescent lamp, 60W, Philips, Taiwan) for photocatalytic reaction, and a light meter (model LX-102, Lutron Electronic Enterprises, Taiwan) was used to record the illumination density. In the dose-dependence experiments, illuminations were carried out for 5 min at a distance of 5 and 15 cm from the lamp, corresponding to the illumination density of 3 × 10^4^, and 5 × 10^2 ^lux (lumen/m^2^)(90 and 10 mW/cm^2^), respectively. In the kinetic analysis experiments, illuminations were carried out for 1, 5, 10, 20, and 40 min at a distance of 5 cm, corresponding to an illumination density of 3 × 10^4 ^lux (90 mW/cm^2^). After illumination, the bacterial solutions were recovered from the 24-well cell culture dishes, and an aliquot of fresh culture medium (250 μl) was used to flush the wells through repeatedly pipetting to further collect the residual bacteria on the wells of the culture dish. The two bacterial solutions were pooled to make a total of 350 μl. The bacterial concentration was determined by the standard plating method immediately after the bacterial collection, and percentage of surviving bacteria was calculated. Polystyrene latex beads were purchased from Sigma-Aldrich (Saint Louis, Mo, USA) and used as negative controls.

### Statistical analysis

All results were calculated from data of three independent experiments. *T*-test was used to assess statistical significance of differences in results of the antimicrobial effects. A *P *value of less than 0.05 (*P *< 0.05) was considered significant. The statistical tests were carried out and output to graphs using the Microsoft Excel (Microsoft Taiwan, Taipei, Taiwan) and SigmaPlot (Systat Software, Point Richmond, CA, USA) software.

## Results

### Electron microscopic and Raman spectroscopic analysis

**Figure 1:**
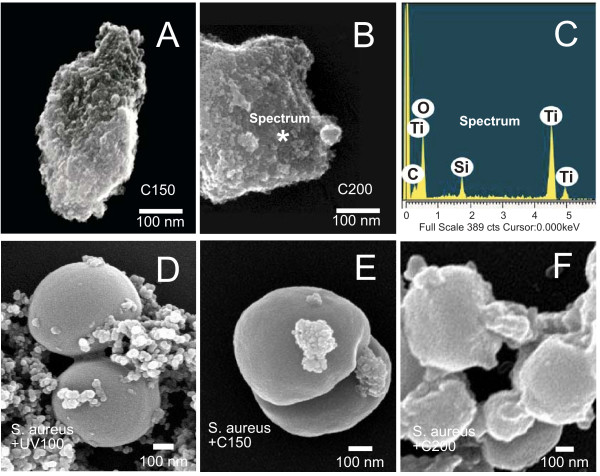
**Scanning electron microscope images of the TiO_2 _nanoparticles**. (A) C150, (B) C200, (C) EDS elemental spectrum of C200, (D) *S. aureus *and UV100, (E) *S. aureus *and C150, and (F) *S. aureus *and C200. Scale bars: 100 nm.

The interaction of the bacteria and TiO_2 _nanoparticles was observed using scanning electron microscope (Fig. 1). Fig. 1A, B depicts the SEM images of the aggregated C150 and C200 TiO_2 _nanoparticles. The sizes appear larger that the dispersed primary particles due to particle aggregations. Fig. 1C is the Energy dispersive x-ray spectroscopy (EDS) that indicates the elemental analysis of the investigated nanoparticles. As shown in the EDS spectrum, the investigated TiO_2 _nanoparticles contain carbons in addition to Ti and oxygen. The carbon contents was estimated to be 1 weight % or 10 atomic % from the EDS spectrum. In Fig. 1D–F, the SEM images revealed the interaction between the tested TiO_2 _samples and the *S. aureus*. As seen in these images, the nanometer-sized TiO_2 _can effectively interact with the bacteria *S. aureus*. However, commercial UV100 TiO_2 _that works as photosensitizer only in the UV range of the light spectrum, the morphology of the bacteria was not affected when interacted with the TiO_2 _upon visible-light illumination (Fig. 1D). For the C150 sample, already some effect was seen on bacterial morphology (Fig. 1E). As to the C200 sample, the morphology of the bacteria was strongly altered due to the interaction with the TiO_2 _under visible-light illumination (Fig. 1F). The SEM investigation showed that C200 sample upon visible-light illumination would spread over the bacterial surface, although bare C200 sample showed aggregation due to their nanometer sizes and strong van der Waals force interaction. This observation is consistent with our bacterial killing test for different strains of bacteria used, and the results will be shown in the following sections.

**Figure 2:**
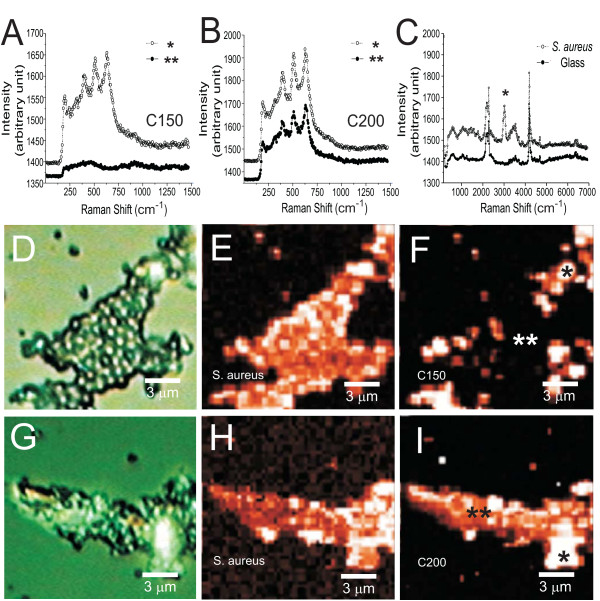
**Raman spectra and confocal Raman mapping of the interaction of *S. aureus *with TiO_2 _nanoparticles**. The Raman spectra of (A) C150, (B) C200 and (C) *S. aureus*. Optical image of the aggregated bacteria *S. aureus *interacting with C150 (D), confocal Raman mapping of the *S. aureus *Raman signals (E) and confocal Raman mapping of C150 (F), optical image of the aggregated bacteria *S. aureus *interacting with C200 (G), confocal Raman mapping of the *S. aureus *signals (H) and confocal Raman mapping of C200 (I).

However, the observation using the scanning electron microscope can only be achieved in high vacuum environment; and the samples required gold coating for imaging. This may cause the complication on the test bacterial samples. To analyze the bacterial samples in a relatively non-invasive way, the bacterial interaction with TiO_2 _was further observed with confocal Raman spectroscopic mapping in ambient. In Fig. 2, the Raman spectra of C150, C200, and the *S. aureus *(Fig. 2A–C) and the spectra of the corresponding positions indicated in the confocal Raman mapping images (Fig. 2E, F and Fig. 2H, I) are shown, respectively. In the Raman spectra, the spectral assignments are; 586, 682 cm^-1 ^for anatase phase; 421, 461 cm^-1 ^for rutile phase of the TiO_2 _crystal structures. The unique Raman peak at ~3000 cm^-1 ^was used as a marker for imaging the position of bacteria *S. aureus *(Fig. 2C). Optical microscopy images show the typical examples of mixed aggregates of C150 and C200 with the *S. aureus *(Fig. 2D, G); and the signal of Raman mapping further reveals the distribution and the position of bacteria or TiO_2 _(Fig. 2E, F and Fig. 2H, I). For the C150 (Fig. 2F), the bright spots indicated the locked C150 Raman signals. It appears randomly across the bacteria *S. aureus *(the bright images in Fig. 2E). For the sample C200, the Raman mapping for both the *S. aureus *and the C200 completely overlapped, suggesting a uniform coverage of the TiO_2 _on the bacteria *S. aureus*. This observation is completely in agreement with the SEM observation (Fig. 1E, 1F). The result indicates C200 sample has better interaction with the observed bacteria *S. aureus*.

### Killing of *S. aureus *by C150 and C200

**Figure 3:**
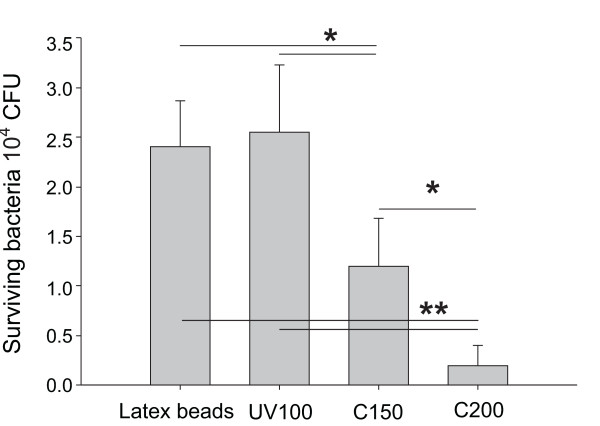
**Bactericidal activity of UV100, C150 and C200 against *S. aureus***. Illumination was carried out at a light density of 3 × 10^4 ^lux for 5 min. * *P *< 0.05, ** *P *< 0.01. Latex beads were used as negative controls.

To compare the bactericidal activities of the TiO_2 _nanoparticles, we mixed 2.5 × 10^4 ^CFU *S. aureus *with 30 μg of UV100, C150, or C200 in 200 μl solutions and irradiated the solutions with 3 × 10^4 ^lux visible-light for 5 min. After irradiation, bacteria solutions were recovered and the number of surviving bacteria was determined by standard plating-out method. Latex beads were used as a negative control. As shown in Fig. 3, C200 exhibited a significantly greater ability to reduce *S. aureus *number compared to latex beads and UV100 (Fig. 3, * *P *< 0.05, ** *P *< 0.01).

**Figure 4:**
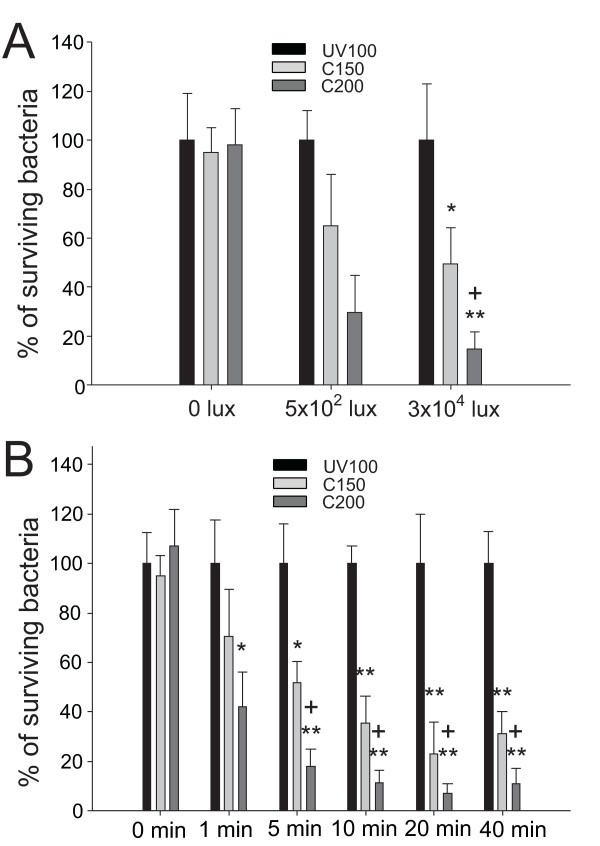
**Dose dependency and kinetics**. Dose dependency (A) and kinetic (B) analyses of the visible-light induced bactericidal activity against *S. aureus *of TiO_2_-related photocatalyst substrates were shown. Illumination was carried out either at different light densities for 5 min (A) or at a light density of 3 × 10^4 ^lux for different times (B). In each illumination condition, the percentages of the surviving bacteria in C150 and C200 groups were normalized to the percentage of the surviving bacteria in the UV100 groups (100%). * *P *< 0.05 and ** *P *< 0.01 compared to the respective UV100 groups. + *P *< 0.05 compared to the respective C150 groups.

To obtain dose dependent and kinetic data for *S. aureus *with C200 substrates, we further analyzed the effects of illumination by visible-light at various time points or at various distances (5 cm, 15 cm, and with different illumination intensities of 3 × 10^4 ^and 5 × 10^2 ^lux) (Fig. 4). The results show that C200 substrates could kill *S. aureus *in minutes when exposed to various degrees of illumination by visible-light (Fig. 4A, B). Even though the bacteria killing efficiency in both C150 and C200 groups were significantly greater than the comparing UV100 groups (Fig. 4A, 4B, ** *P *< 0.01; * *P *< 0.05), C200 still has superior performance when compared to C150 groups (Fig. 4A, 3 × 10^4 ^lux groups; 4B, 5 min to 40 min groups, + *P *< 0.05). The observation is in agreement with both the SEM and Raman observations that C200 sample exhibited distinct performance when interacting with the tested bacteria.

### Bacteria-killing experiment for other pathogens

**Figure 5:**
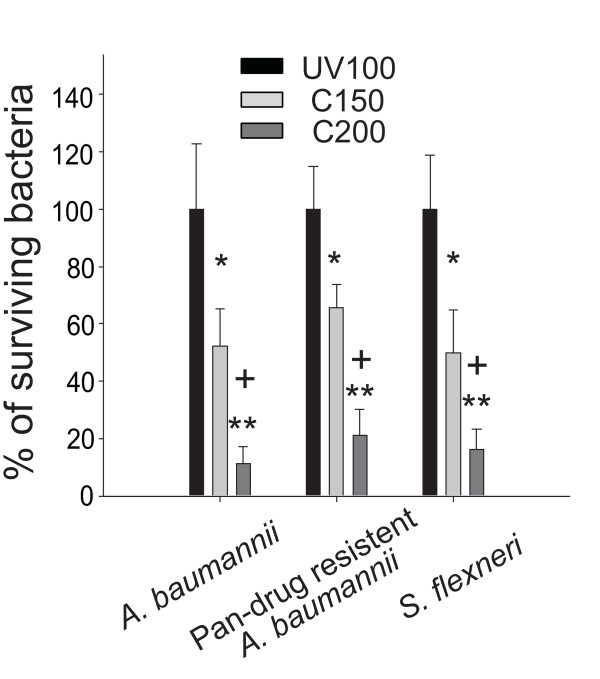
**Pathogen analysis**. For each pathogen, the percentage of surviving bacteria on the C150 and C200 substrates was normalized to that on the UV100 substrates. Illumination was performed at light density of 3 × 10^4 ^lux for 10 min. * *P *< 0.05 and ** *P *< 0.01 compared to the respective UV100 groups. + *P *< 0.05 compared to the respective C150 groups.

Bactericidal activities of C150 and C200 on other human pathogens including *A. baumannii*, pan-drug resistant *A. baumannii *and *S. flexneri *were also examined. C200 demonstrated significantly higher effectiveness in killing of all tested bacteria, as compared to C150 and UV100 (Fig. 5).

## Discussion

Urbanization, population growth and heavy traveling enable infectious diseases to quickly spread worldwide from one local area. Photocatalyst has the potential for use in a variety of settings to reduce the transmission of pathogens in public environments. The emergence of increasingly virulent and antibiotic resistant pathogens in hospital settings [1,3] provides another motivation for the development of alternative disinfection approaches using photocatalyst. There are several advantages to use the visible-light responsive photocatalyst such as titania. First, for safety consideration, visible light is a relatively safer light source as compared to UV irradiation [33]. Exposure to UV light at the necessary levels, would cause great damage of skin and eye tissues for humans [33-35]; thus limiting the use of conventional UV activated TiO_2 _substrates in environments where humans would be exposed. The visible-light activated photocatalyst offers a perfect alternative for use as a disinfectant in public areas. Second, because TiO_2 _is a chemically stable and inert material, it could continuously exert antimicrobial action when illuminated by light. Third, the bactericidal activity can be switched on and off or modulated by controlling the light intensity. In addition, from efficiency point of view, commercial titania absorbs only the UV range estimated 2–3% of solar light impinging on the Earth's surface [36] when used for an outdoor setting. The advantages of the visible-light responsive photocatalyst might be complementary to existing disinfectants and provide the potential for developing a variety of alternative antimicrobial applications. To extend the light-absorption into visible-light range, doping with transition ions and/or anions (negative ions) is a commonly used method. By which it creates intra-band gap states close to the conduction or valence band edges that induces visible-light absorption at the sub-band gap energies [9,36]. In some cases, the doped materials also are able to inhibit the charge recombination, thereby increase the photocatalytic activity [36-39]. Using such approach, many studies have shown to develop titania photocatalyst with antimicrobial activity in the visible-light range [13-15,38-46]. In these studies, anions such as sulfur, nitrogen and carbon, and metal ions such as neodymium, tungsten, and platinum were used for doping titania. The photocatalytic- and antimicrobial-performance of these dopants are different because the various roles of the doped materials in trapping electrons and/or holes on the surface.

Besides photocatalytic activity, there are still other unidentified factors affect the antimicrobial activity. For example, catalysts may have similar photocatalytic activity but with different bactericidal performance as observed in the study using titania-coated nickel ferrite [40]. Anatase-titania-coated nickel ferrite and brookite-titania-coated nickel ferrite have a similar photocatalytic reaction rate, while the former one has a superior bacterial-inactivation response [40]. This indicates the physical properties such as bacterium-catalyst interactions might influence the antimicrobial outcome.

To analyze the influence of bacterium-catalyst interactions on the antimicrobial performance, we used C150 and C200 titania catalysts. Previously we found that both C150 and C200 samples have a similar visible light absorption pattern [12]. C200 nanocrystals, however, contain mixed anatase and rutile phases that resulted in interface states in the mixed surface energy structures, as compared with uniform anatase phase structure of C150 nanocrystals [12]. The distinct bacterial interaction behaviors of the C150 and C200, as observed in the SEM and Raman mapping in this study, presumably are attributed to the existence of different structural complexity. The interactions between TiO_2 _with biomolecules were rarely discussed. It was shown that various sol-gel treatments can change the property of TiO_2 _surfaces [47,48]. It was also shown that different TiO_2 _crystal surfaces indeed have different affinities toward cellular protein fibronectin [49]. In addition, carbon-coated TiO_2 _samples showed high affinity and high photoactivity towards organic compound methylene blue [48,50]. Since bacterial surfaces express various organic components and proteins, it is not surprising that the bacteria would have a preferential interaction with specific catalyst. Using scanning electron microscopy and confocal Raman mapping techniques, here we successfully demonstrated that better bacterial-interaction is associated with better pathogen-killing performance when C200 samples were tested in bactericidal experiments.

In conclusion, we found that by generating mixed-phase TiO_2 _nanocrystals, the antibacterial activity of carbon-containing photocatalyst was significantly enhanced; and the photocatalysts can be used in the visible light settings. Although the bactericidal activity remains to be further improved and optimized, the unique property of C200 to interact with bacteria might provide a new perspective for developing more effective antibacterial photocatalysts.

## Competing interests

The authors declare that they have no competing interests.

## Authors' contributions

CLC carried out the Raman spectra and confocal Raman mapping, and participated in its design. DSS and WCC carried out the photocatalysis experiments. YHT participated in the synthesis of photocatalysts. HCH participated in the SEM analysis. JBW and PHC participated in the confocal Raman mapping. JHC, PJT, NTL and MSU participated in the analysis of pathogenic bacteria. HHC conceived of the study, and participated in its design, coordination and drafted the manuscript. All authors read and approved the final manuscript.
